# Attitudes of the Population Toward Vaccines During the COVID‐19 Pandemic: The PROACTIVE Study

**DOI:** 10.1111/phn.13561

**Published:** 2025-04-04

**Authors:** Michela Calzolari, Mariarosaria Gammone, Daniela Cattani, Giulia Ottonello, Giuseppe Aleo, Loredana Sasso, Milko Zanini, Gianluca Catania, Annamaria Bagnasco

**Affiliations:** ^1^ Department of Health Sciences University of Genoa Genoa Italy; ^2^ Fondazione IRCCS Istituto Neurologico Carlo Besta Milan Italy; ^3^ Department of Biomedical Sciences Humanitas University Pieve Emanuele Milan Italy; ^4^ Ingram School of Nursing McGill University Montréal QC Canada; ^5^ Research Fellow Faculty of Nursing & Midwifery Royal College of Surgeons in Ireland Dublin Ireland

**Keywords:** attitudes, COVID‐19 vaccines, health knowledge, practice, preventive health services, public health nursing, vaccination, vaccine hesitancy

## Abstract

**Background:**

Vaccination is a vital strategy to prevent infectious diseases and reduce mortality globally. However, vaccine hesitancy threatens these efforts, especially highlighted during the COVID‐19 pandemic. Understanding factors influencing vaccination decisions is crucial for improving public health strategies.

**Objective(s):**

To investigate the attitudes of the Italian general population toward mandatory (e.g., HBV or tetanus) or recommended (e.g., influenza, HPV, or meningococcus) vaccinations, factors influencing vaccine uptake, and risk perceptions related to COVID‐19.

**Design:**

A cross‐sectional descriptive study using the *PROACTIVE Survey* questionnaire.

**Sample:**

The study included 411 participants aged 18–98 years from the general Italian population, recruited via convenience and snowball sampling in June 2022.

**Measurements:**

Data included sociodemographic characteristics, adherence to vaccinations, COVID‐19 experiences, preventive behaviors, and individual risk perceptions. Inferential statistics included Pearson's *r* correlation, *t*‐test, and analysis of variance (ANOVA) to explore correlations and differences.

**Results:**

Adherence to preventive measures positively correlated with risk perceptions (*r* = 0.358, *p *< 0.001). Females, older individuals, and those with chronic conditions showed higher adherence to preventive behaviors. Previous adherence to vaccines correlated with greater COVID‐19 preventive behaviors (*r* = 0.124, *p *= 0.012).

**Conclusions:**

Age, gender, risk perceptions, and chronic conditions significantly influenced vaccination attitudes and preventive measures. These findings underscore the need for tailored public health strategies, especially in post‐pandemic contexts, to address vaccine hesitancy and improve vaccination campaigns.

## 1 Background

Vaccination is considered an effective approach to prevent infection and reduce mortality of many infectious diseases. It is estimated that it currently prevents 2–3 million deaths a year and that a further 1.5 million could be avoided if global coverage of vaccinations were improved (WHO, 2019).

Already in 2017, the World Health Organization (WHO) underlined that the stated vision of the Decade of Vaccines (2011–2020) was for “*a world in which all individuals and communities enjoy lives free from vaccine‐preventable diseases”* (WHO, 2019). To date, the emphasis on immunization remains a major issue. In fact, one of the 17 United Nations’ Sustainable Development Goals, (UN, 2021) is to *“ensure healthy lives and promote well‐being for all at all ages”*, and includes the *“access to safe, effective, quality and affordable essential medicines and vaccines for all”*.

Despite evidence for the effectiveness of vaccination, according to the WHO (2019) the phenomenon of vaccine hesitancy, defined as the reluctance or refusal to vaccinate despite the availability of vaccines, threatens to reverse progress made in tackling vaccine‐preventable diseases.

The 2018 European Parliament resolution on vaccine coverage rates in Europe, already urged member states to ensure sufficient vaccination of healthcare workers, to adopt effective measures against misinformation, and to implement measures to improve access to medicines. It also asked the European Commission to facilitate a more harmonized vaccination schedule across Europe (European Parliament, 2018).

Some studies evaluated risk factors associated with low adherence to vaccination. For example, a study conducted in the USA evaluated adherence to vaccine for Hepatitis and showed that adherence and completion rates varied by patient demographic, socioeconomic, and clinical characteristics. Overall, younger age, Black/Hispanic ethnicity, having comorbidities, and lower household income or lower educational status tended to be associated with lower vaccine adherence and completion rates (LaMori et al. 2022).

To date, the WHO Regional Office for Europe recently celebrated the 2023 European Immunization Week with the aim of raising awareness on the importance of immunization to prevent diseases and protect life, and of improving vaccine uptake, in the context of European Immunization Agenda 2030 (WHO, 2023). The key messages of this initiative focus on re‐engaging the population on the importance of timely routine vaccination and on catching up on any missed or postponed vaccines and continue to emphasize the need for COVID‐19 vaccination, especially among vulnerable groups (WHO, 2023), such as older people, people with chronic medical conditions, healthcare workers, and residents of long‐term care facilities. Recommendations for these at‐risk groups are already standard in most countries, for example, for the seasonal influenza vaccination. Regarding this vaccination, a survey published by the European Centre for Disease Prevention and Control in 2018, reported the influenza vaccination coverage rates (VCRs) among at‐risk groups in the 19 EU/EEA (European Union/European Economic Agreement) Members where these results were known, from 2015 to 2017. For example, in older population groups VCRs varied from 2% to 72.8% in the season 2016–2017, with 47.1% median VCR. The highest VCRs were reported by the United Kingdom, which almost achieved the European target of 75%.

In chronic medical conditions’ group (e.g., pulmonary diseases, cardiovascular diseases, renal diseases, metabolic disorders, and immunosuppression due to diseases or treatments), VCRs were considerably lower than the older population groups in most Member States and did not meet the European targets, except for Ireland and the United Kingdom (European Centre for Disease Prevention and Control 2018).

In March 2023, the coronavirus (COVID‐19) pandemic resulted in more than 760 million confirmed worldwide cases and over 6.8 million deaths. (WHO, 2023).

Regarding COVID‐19 vaccines, it became clear that the population's hesitation on receiving the vaccine would determine the success or failure of the proposed prevention strategies. Although many studies showed evidence of vaccine effectiveness in reducing the number of COVID‐19 cases and especially of severe forms of disease (Graña et al. 2022), according to current data by WHO, in Europe only 65.35% of the population has been fully vaccinated with the complete immunization cycle with differences in vaccine adherence across Europe. In almost all Western European countries vaccination coverage is over 70% while for Eastern countries it is lower, with a minimum of 25.79% in Bosnia and Herzegovina (WHO, 2023). According to the WHO, all countries should continue to work toward vaccinating at least 70% of their populations, prioritizing the vaccination of 100% of healthcare workers and of the most vulnerable groups, including people who are over 60 years of age and those who are immunocompromised or have underlying health conditions (WHO, 2023). To do so, it is essential to understand the hesitancy phenomenon and what strategies are effective in reducing it. A study conducted on general population in the UK, before COVID‐19 vaccine campaign started, found that the most powerful predictors of vaccine hesitancy were attitudes to vaccines in general and conspiracy suspicions with regard to coronavirus (Allington et al. 2023). In this context, healthcare professionals could positively influence the population's decisions about vaccination, through health education and trusting relationship with citizens (Jaca et al. 2018; Lyon et al. 2023; Moola et al. 2021; Kaufman et al. 2013).

In Italy, it has been estimated that, between January 2021 and January 2022, COVID‐19 vaccination directly prevented approximately 8 million infections, over 500,000 hospitalizations, more than 55,000 intensive care unit admissions, and around 150,000 deaths (Istituto Superiore di Sanità 2022). The national COVID‐19 vaccination campaign started in December 2020, excluding children aged 0–4 years. In addition to the primary vaccination series, consisting of two doses or a single dose depending on the vaccine, two booster doses were authorized in 2022. In both instances, booster doses were initially recommended for individuals aged ≥80 years (irrespective of health status), residents of long‐term care facilities, and individuals aged 60–79 years with risk factors for severe COVID‐19 progression. By the end of October 2022, 85.8% of the Italian population had received at least one vaccine dose, with 77.6% having received their last dose more than 180 days prior, and only 8.3% within the previous 180 days; 14.1% of the population remained unvaccinated (Istituto Superiore di Sanità 2022). As of the same date, the susceptible population, calculated by excluding individuals with a documented SARS‐CoV‐2 infection in the preceding 90 days and those who had died, was estimated at 54,558,793 individuals (Istituto Superiore di Sanità 2022).

Recent literature reviews have examined the factors associated with vaccine hesitancy in Italy (Ferrara et al. 2023; Primieri et al. 2023), highlighting the complex interplay of behavioral, cognitive, and perceptual determinants influencing COVID‐19 vaccination acceptance. A positive attitude toward preventive health behaviors (such as wearing masks, adherence to medical treatment, participation in influenza vaccination programs, and cancer screening) was significantly correlated with higher vaccine uptake (Primieri et al. 2023). Additionally, confidence in science, medicine, healthcare institutions, and healthcare professionals, as well as trust in vaccines in general, emerged as key factors promoting vaccination acceptance. Perceived risk of COVID‐19 also played a crucial role, with several studies reporting a significant association between heightened risk perception and greater willingness to vaccinate (Primieri et al. 2023). Furthermore, beliefs regarding the vaccine's safety, efficacy, and overall utility, along with personal or vicarious experiences of severe COVID‐19 outcomes among family members, friends, or acquaintances, were positively linked to vaccine acceptance (Primieri et al. 2023). Conversely, vaccine hesitancy was associated with perceived insufficient information, concerns about safety and adverse effects, and overall low confidence in COVID‐19 vaccination (Ferrara et al. 2023; Primieri et al. 2023).

For these reasons, it is essential for healthcare professionals to understand what are the factors that influence population's decisions regarding whether to adhere to vaccinations. In particular, reflecting in a post‐COVID‐19 context, where the vaccination campaign particularly had an impact on the population, can help to better understand this phenomenon and may provide useful insights into the planning of effective future vaccination campaigns.

### 1.1 Aim

This study was conducted during the first 2 weeks in June 2022, and it aimed at identifying the attitudes of the general population toward vaccination against a series of diseases and related factors, the perception of the risk related to the COVID‐19 disease, the preventive measures adopted by citizens during the COVID‐19 pandemic.

## 2 Methods

### 2.1 Design

This was a cross‐sectional descriptive study. It was part of a larger study called the “Population and healthcare workers PeRspectives and mOtivational factors Towards covId‐19 VaccinE” (PROACTIVE) study.

### 2.2 Ethical Considerations

This study was approved by the Liguria Regional Ethics Committee (Reg. N. 207/2021–DB id 11397). The study was conducted in compliance with the Declaration of Helsinki (Fortaleza version, 2013) and in accordance with current regulations on clinical trials and good clinical practice. The study promoter ensured the protection of participants' sensitive personal data in line with European legislation (EU GDPR Regulation 2016/679). Participation in the study was entirely voluntary, with participants having the right to withhold consent or withdraw at any stage of the study. All data were anonymized to safeguard participants’ privacy in accordance with applicable privacy regulations (EU GDPR Regulation 2016/679).

### 2.3 Sample

For this study, the Italian general population was defined as all adults who were not healthcare workers, and who accessed the COVID‐19 vaccination hub of a large hospital in Northern Italy. Therefore, participants were recruited through convenience and snowball sampling. Data collection was started at the beginning of June 2022 and lasted 15 days. At the time of the study, in Italy COVID‐19 vaccination was mandatory and those who refused had to pay a fine. Therefore, we assumed that also most of those who were hesitant to vaccination, accessed the vaccination hub independently from their personal belief.

In this case, participants were recruited at the time they were accessing the vaccination hub to undergo vaccination. At that time, they were given the questionnaire to fill out online via QR code. Since the respondents enrolled with this modality were not representative of the study population, a parallel snowball sampling was conducted. In this case, participants were recruited through personal acquaintances, and the link to fill out the questionnaire was provided to them via email or social media.

### 2.4 Measures

The survey was composed of the PROACTIVE_Survey questionnaire. The instrument was developed based on the study by Schwarzinger et al. (2021a) on vaccine hesitancy in a representative working‐age population in France. We validated the instrument in Italian following the EORTC guidelines. The validated Italian version showed good validity and reliability (Cronbach *α* = 0.96; S‐CVI = 0.98).

The instrument is composed of 32 items with closed‐ended questions or 4‐point Likert scale (0 = A lot/always; 1 = Quite a lot/often; 2 = Not much/sometimes; 3 = Nothing/never, depending on the question), investigates three components (sociodemographic characteristics, behaviors, and attitudes) and is articulated in five sections. Section 1 explored the sociodemographic characteristics (eight questions), such as age, sex, and educational level.

Section 2 explored the risk factors for severe forms of COVID‐19 disease (seven questions) and previous adherence to other vaccines (three questions), by asking questions such as “*In the past, have you performed seasonal flu vaccination?*”. Section 3 explored the experience with COVID‐19 (four questions), by asking questions such as *“Have you performed a test to diagnose COVID‐19 infection?”*. Section 4 explored the preventive measures implemented in addition to vaccination against COVID‐19 (six questions), by asking questions such as *“Do you wash your hands or use hand gel after using public transport?”*. Section 5 explored the study participants' individual risk perceptions (four questions), by asking questions such as “*During the period of isolation at home due to the COVID‐19 emergency, how worried were you about falling ill with COVID‐19*?”.

### 2.5 Analytic Strategies

We used descriptive statistical indices such as mean and standard deviation or percentage to describe the various sections of the survey. After checking the normal distribution of the data, we used inferential statistics to identify correlations between the various sections of the survey (Pearson's *r*) and the differences between participants’ characteristics and the various sections’ scores (*t*‐test, ANOVA).

## 3 Results

### 3.1 Section 1. Demographic Characteristics

In total, the participants were 411. The mean age was 36.2 years, with a minimum of 18 years and a maximum of 98 years. Most of the participants were female (*n* = 263, 64%), lived in an urban area (*n* = 345, 83.9%), had a high school diploma (*n* = 273, 66.4%), and were students (*n* = 173, 42.1%) or employees (*n* = 157, 38.2%) (Table 1).

**TABLE 1:** Demographic characteristics.

	*n* (%)	Mean (SD)
Age		36.2 (17.8)
**Gender**		
Male	148 (36)	
Female	263 (64)	
Other	0 (0)	
**Level of education**		
Primary school	8 (1.9)	
Middle school	47 (11.4)	
High school	273 (66.4)	
University/Post‐degree	83 (20.2)	
Number of adults (aged 18 and older) living together		2.09 (2.13)
Number of children (under the age of 18) living together		0.38 (0.70)
**Occupational status**		
Unemployed	21 (5.1)	
Employee	157 (38.2)	
Self‐employed	31 (7.5)	
Student	173 (42.1)	
Retired	29 (7.1)	
**Area of residence**		
Urban	345 (83.9)	
Rural	66 (16.1)	
**Work in contact with the public**		
Yes	225 (54.7)	
No	186 (45.3)	

### 3.2 Section 2. Risk Factors and Previous Adherence to Other Vaccines

Many participants were smokers (26.8%) or used to smoke in the past (21.9%), 9.2% of the participants suffered from hypertension, 16.3% suffered from chronic diseases, in particular asthma (20.9%) and diabetes (16.4%). Most of participants had a normal weight (60%), but a large proportion were overweight or obese (31%) (Table 2).

**TABLE 2:** Descriptive analysis of Sections 2, 3, 4, and 5.

		*n* (%)	Mean (SD)
**Section 2. Risk factors and previous adherence to other vaccines**			
Vaccination behavior in the past			
In the past, have you performed seasonal flu vaccination?	Always	41 (10)	
	Often	39 (9.5)	
	Sometimes	90 (21.9)	
	Never	241 (58.6)	
In the past, have you performed mandatory vaccinations?	Always	363 (88.3)	
	Often	27 (6.6)	
	Sometimes	19 (4.6)	
	Never	2 (0.5)	
In the past, have you performed the recommended vaccinations?	Always	175 (42.6)	
	Often	81 (19.7)	
	Sometimes	91 (22.1)	
	Never	64 (15.6)	
**Total score vaccination behavior**			5.43 (1.79)
**Risk factors for severe forms of COVID‐19**			
Are you currently, or do you think you are, pregnant?	Yes	6 (1.5)	
	No	296 (72)	
	Not applicable	109 (26.5)	
Compared to tobacco smoking, what is your situation?	I currently smoke	110 (26.8)	
	I never smoked	211 (51.3)	
	I have smoked in the past	90 (21.9)	
Weight (kg)			67.29 (14.17)
Height (cm)			169.09 (8.54)
Do you suffer from hypertension?	Yes	38 (9.2)	
	No	373 (90.8)	
Do you suffer from other chronic conditions?	Yes	67 (16.3)	
	No	344 (83.7)	
If YES, which of the following?	Diabetes	11 (16.4)	
	Asthma	14 (20.9)	
	Chronic respiratory diseases	2 (3)	
	Chronic vascular diseases	5 (7.5)	
	Chronic heart diseases	5 (7.5)	
	Liver disease	0 (0)	
	Hematologic/oncologic diseases	4 (6.0)	
	Other	26 (38.8)	
**Section 3. Experience with COVID‐19**			
Have you experienced symptoms attributable to COVID‐19 infection?	Yes	200 (48.7)	
	No	211 (51.3)	
Have you performed a test to diagnose COVID‐19 infection?	Yes	376 (91.5)	
	No	35 (8.5)	
Do you know anyone who has contracted COVID‐19?	Yes, with hospitalization	177 (43.1)	
	Yes, without hospitalization	228 (55.5)	
	No	6 (1.5)	
Have you had contact with people affected by COVID‐19?	Yes	293 (71.3)	
	No	59 (14.4)	
	I don't know	59 (14.4)	
**Section 4. Preventive measures implemented in addition to vaccination against COVID‐19**			
Do you wash your hands when you get home?	Always	292 (71)	
	Often	88 (21.4)	
	Sometimes	29 (7.1)	
	Never	2 (0.5)	
Do you wash your hands or use hand gel after using public transport?	Always	230 (56)	
	Often	134 (32.6)	
	Sometimes	31 (7.5)	
	Never	16 (3.9)	
Do you use hand gel when entering a shop, supermarket or mall?	Always	150 (36.5)	
	Often	156 (38)	
	Sometimes	84 (20.4)	
	Never	21 (5.1)	
Do you wear a mask in any closed public place?	Always	168 (40.9)	
	Often	129 (31.4)	
	Sometimes	95 (23.1)	
	Never	19 (4.6)	
Do you wear a mask in any open public place (e.g., parks, gardens, streets)?	Always	24 (5.8)	
	Often	48 (11.7)	
	Sometimes	175 (42.6)	
	Never	164 (39.9)	
Do you respect the recommended social distancing rules?	Always	118 (28.7)	
	Often	148 (36)	
	Sometimes	110 (26.8)	
	Never	35 (8.5)	
**Total score preventive measures**			11.9 (3.55)
**Section 5. Individual risk perceptions**			
During the period of isolation at home due to the COVID‐19 emergency, how worried were you about falling ill with COVID‐19?	A lot	101 (24.6)	
	Quite	191 (46.5)	
	Little	98 (23.8)	
	Not at all	21 (5.1)	
In the period before isolation at home due to the COVID‐19 emergency, how worried were you about falling ill with COVID‐19?	A lot	65 (15.8)	
	Quite	139 (33.8)	
	Little	131 (31.9)	
	Not at all	76 (18.5)	
Right now, how worried are you about getting COVID‐19?	A lot	15 (3.6)	
	Quite	76 (18.5)	
	Little	243 (59.1)	
	Not at all	77 (18.7)	
How worried are you about getting COVID‐19 this fall?	A lot	23 (5.6)	
	Quite	103 (25.1)	
	Little	219 (53.3)	
	Not at all	66 (16.1)	
**Total score Individual risk perceptions**			5.65 (2.59)

The overall score for attitudes toward vaccines in general, obtained by calculating the total average of the three questions in Section 2 (the above‐mentioned scores were applied for each question) was 5.43 (SD 1.79; Range: 0–9). In particular, participants showed a greater adherence to the mandatory and recommended vaccinations, but a lower level of adherence to the flu vaccination (Table 2).

### 3.3 Section 3. Experience With COVID‐19

Almost half of the participants had experienced symptoms attributable to COVID‐19, but 91.5% had taken a COVID‐19 test. Almost all the participants knew someone who had tested positive for COVID‐19, and many knew someone who was hospitalized as a result (43.1%). Most participants (71.3%) had at least one contact with a COVID‐19 patient (Table 2).

### 3.4 Section 4. Preventive Measures Implemented in Addition to Vaccination Against COVID‐19

The overall score of this section was 11.9 (SD 3.5; range 0–18). In particular, the most used preventive measures were washing hands at home and after the use of public transport. The least used was the use of masks outside. A small proportion of the participants (8.5%) never respected the social distancing measure, but the difference between those who respected it always, often, or sometimes was minimal (Table 2).

### 3.5 Section 5. Study Participants' Individual Risk Perceptions

In this section, participants were asked how worried they were about contracting COVID‐19 at different stages of the pandemic. The most frightening moment was during the period of isolation at home during the first wave of the pandemic, where most participants were “a lot” (24.6%) or “quite” (46.5%) worried. On the contrary, the least frightening moment was the one when they were currently completing the survey. In fact, most of the participants (59.1%) were “little worried” about getting COVID‐19 (Table 2).

### 3.6 Intercorrelations Between Sections of the PROACTIVE_Survey

The overall scores of the different sections of the questionnaire were tested for correlations. Significant positive correlation was found between Section 4 (preventive measures implemented in addition to vaccination against COVID‐19) and Section 5 (participants’ individual risk perception) (*r* = 0.358, *p* < 0.001) (Table 3). We then analyzed these two sections with different items from the other sections for correlation. For these analysis, Section 4 and 5 were therefore merged (Section 4–5).

**TABLE 3:** Correlations between the different sections of the PROACTIVE survey.

		Pearson's *r*	*t*‐test	*p* value	95% CI
Score Section 4		0.358		<0.001*	0.27 to 0.44
Score Section 5					
Section 4–5		0.220		<0.001	0.13 to 0.31
Age					
Respect of social distancing (Spearman's *ρ*)		0.166		<0.001*	0.04 to 0.23
Age					
Section 4–5			−6.14	<0.001*	−0.84 to −0.42
Gender	Male versus Female				
Section 4–5					
Occupational status					
	Unemployed versus Self‐employed	5.21		0.002*	
	Unemployed versus Employee	2.58		0.166	
	Unemployed versus Student	4.35		0.002*	
	Unemployed versus Retired	1.93		0.656	
	Employee versus Self‐employed	2.63		0.056	
	Employee versus Student	1.77		0.012*	
	Employee versus Retired	−0.66		0.965	
	Self‐employed versus Student	−0.86		0.899	
	Self‐employed versus Retired	−3.29		0.079	
	Student versus Retired	−2.42		0.108	
Section4–5					
Level of education					
	Primary school versus Middle	−1.48		0.808	
	Primary school versus High school	−1.64		0.702	
	Primary school versus University/post‐degree	−2.50		0.431	
	Middle school versus High school	−0.15		0.998	
	Middle School versus University/post‐degree	−1.02		0.734	
	High school versus University/post‐degree	−0.87		0.576	
Section 4–5					
Area of residence					
	Urban versus Rural		−0.72	0.473	−0.36 To 0.17
Section 4–5			2.69	0.007*	0.09 to 0.63
Chronic disease	Yes versus No				
Section 4–5					
Risk factors					
	I currently smoke versus I never smoked	0.10		0.984	
	I currently smoke versus I have smoked in the past	−0.20		0.957	
	I never smoked versus I have smoked in the past	−0.31		0.885	
Section 4		0.124		0.012*	0.03 to 0.22
Vaccination behavior					

A positive correlation was found between the age of participants and the Section 4 (preventive measures implemented in addition to vaccination against COVID‐19)—Section 5 (participants’ individual risk perception) regarding participants’ individual risk perception and the adoption of preventive measures (*r* = 0.220; *p* < 0.001), in particular, the respect of social distancing measure (*ρ* = 0.166; *p* < 0.001) (Table 3). Always regarding Section 4 (preventive measures implemented in addition to vaccination against COVID‐19)—Section 5 (participants’ individual risk perception), significant differences were found regarding gender, with females generally having a greater adherence to preventive measures and greater individual risk perception (*p* < 0.001) (Table 3). Significant differences were also found between the occupational states: self‐employed and students generally had a minor adherence to preventive measures and minor individual risk perception, as opposed to the unemployed and employee (Table 3). No correlations were found between Section 4 (preventive measures implemented in addition to vaccination against COVID‐19)—Section 5 (participants’ individual risk perception) and the level of education nor the area of residence.

Previous adherence to other vaccines was found to be positively correlated to Section 4 (*r* = 0.124, *p* = 0.012) (Table 3). Regarding the presence of chronic diseases, those who were affected by a chronic disease had a greater adherence to preventive measures and greater individual risk perception (*p* = 0.007) (Table 3). No correlations were found between Section 4 (preventive measures implemented in addition to vaccination against COVID‐19)—Section 5 (participants’ individual risk perception) and participants’ risk factors.

## 4 Discussion

This study showed that preventive measures against COVID‐19 were significantly associated with participants’ individual risk perceptions. Both age and gender were found to be significant factors influencing the perception of risk and adherence to preventive measures, with a greater adherence to preventive measure and individual risk perception in females. Significant associations were also found between previous adherence to other vaccines and preventive measures implemented in addition to vaccination against COVID‐19. Moreover, those who were affected by a chronic disease were found to have a greater adherence to preventive measures and greater individual risk perception.

The association between preventive measure against COVID‐19 and previous vaccine adherence that was found in our study seems unique. To the authors’ knowledge, no other studies were found reporting this association. We found that a greater implementation of preventive measures against COVID‐19 was also correlated to individual risk perception. Risk perception was found to be of great influence toward vaccine adherence in several studies, both regarding specifically the COVID‐19 vaccine and other vaccination campaigns (Allington et al. 2023; Ferrara et al. 2023; Truong et al. 2022; Karafillakis and Larson 2017; Schmid et al. 2017). In this context, it is essential to underline the key role of nurses in the public health area in fostering vaccine confidence and promoting adherence through effective health education. Their expertise in patient communication and education is essential for addressing misconceptions, enhancing risk awareness, and ultimately improving vaccination uptake within communities.

The association between age and attitude toward vaccination is noteworthy. We found that as age increased, hesitancy to vaccination against COVID‐19 decreased. This association has been investigated by many other studies, which also highlighted a negative correlation between age and hesitancy toward vaccination (Garza, Leibensperger, and Bonnevie 2023; Kafadar et al. 2022; Schwarzinger et al. 2021b). However, a systematic review on barriers to influenza vaccination (Schmid et al. 2017) reported inconclusive results regarding the influence of age on this issue.

Therefore, this aspect should be further investigated to improve future vaccination campaigns personalizing interventions according to age.

In our study we also found that females were more likely to respect preventive measures for COVID‐19. Schwarzinger et al. (2021b) in line with the results of the systematic review by Kafadar et al. (2022), reported a correlation with female gender and hesitancy to vaccine. These results may be explained in future studies by exploring what appears to be a tendency of females to be more attentive to non‐pharmacological preventive measures while exhibiting greater hesitancy toward pharmacological interventions.

Regarding the association between hesitancy toward other vaccines and hesitancy toward the vaccine against COVID‐19, it is confirmed by the results of other studies investigating this relationship (Garza et al. 2023; Primieri et al. 2023; Schwarzinger et al. 2021b; Kafadar et al. 2022). Although these vaccines have distinct characteristics and histories, the correlation in hesitancy toward both may prompt researchers to engage in critical analyses and further explore this phenomenon to identify common factors and develop increasingly effective strategies to address vaccine hesitancy.

We found that occupational status and level of education did not influence adherence to the COVID‐19 vaccine. These results are in contrast with the results obtained from other studies, which highlighted that the most disadvantaged populations in terms of economic and educational levels had lower levels of adherence to vaccinations (Garza et al. 2023; Kafadar et al. 2022). This might be because our sample was quite homogeneous, so most participants could have had about the same level of access to information and education.

Having a chronic condition, which in our study was associated with a greater adoption of preventive measures, was positively associated with different kinds of vaccination, including the COVID‐19 vaccine, similarly to other studies (LaMori et al. 2022; Schwarzinger et al. 2021b; Wang et al. 2021).

This is an important result since chronic conditions also constitute a recommendation factor for most of the vaccination campaigns (European Centre for disease prevention and control 2018).

### 4.1 Strengths and Limitations

Our study provided an important overview of the population's attitudes toward vaccination against a series of diseases and related factors. The study was conducted in a post‐pandemic context, which enriched the results prior to that period. A limitation of this study was that the individuals included in the study were primarily convenience sampled and snowballed from people accessing a COVID‐19 vaccination hub, thus not fully representing the Italian general population. In addition, this recruitment approach did not enable us to calculate the response rate. However, considering the size of the sample included in our study, it was possible to gain a good initial understanding of their perceptions about vaccinations.

## 5 Conclusions

Our study reinforced the available scientific evidence around vaccination. In particular, we highlighted that factors such as gender, age, perceived risk, and chronic diseases may contribute to positive attitudes toward both vaccination and preventive strategies. In the post‐pandemic context in which we find ourselves, it is essential to consider the recent experience and reflect on the future.

Although the importance of vaccination and the phenomenon of vaccine hesitancy have been known for a long time, COVID‐19 has made the issues related to this phenomenon and to ineffective vaccination campaigns even more evident.

In the perspective of developing more effective strategies to increase vaccination adherence and combat the spread of infectious, and potentially preventable, diseases, it is crucial to consider the factors identified in the present study. In this regard, the key role of nurses in public health cannot be underestimated. Nurses are pivotal in fostering vaccine confidence and promoting adherence by providing effective health education. Their expertise in patient communication and education is essential for addressing misconceptions, enhancing risk awareness, and ultimately improving vaccination uptake within communities. By actively engaging with individuals and offering clear, evidence‐based information, nurses play a critical role in shaping positive health behaviors and ensuring the success of vaccination campaigns.

## Author Contributions

All listed authors meet the authorship criteria, and all authors agree with the content of the manuscript. All authors have: (1) made substantial contributions to conception and design, or acquisition of data, or analysis and interpretation of data, (2) been involved in drafting the manuscript or revising it critically for important intellectual content, (3) given final approval of the version to be published. Each author has participated sufficiently in the work to take public responsibility for appropriate portions of the content; and (4) agreed to be accountable for all aspects of the work in ensuring that questions related to the accuracy or integrity of any part of the work are appropriately investigated and resolved. In particular: L.S., A.B., G.A., M.Z., and G.C. made a substantial contribution to the conception of this paper. G.C., M.C., M.G., D.G., and G.O. were substantially involved in the design, data acquisition, analysis, and interpretation. M.C., G.C., G.A., and G.O. were mainly involved in drafting and editing the manuscript. G.C., M.Z., L.S., and A.B. contributed to revising it critically for important intellectual content.

## Conflicts of Interest

All the authors declare that they have no conflicts of interest concerning the research, authorship, and/or publication of this article.

## Data Availability

The data that support the findings of this study are available from the corresponding author upon reasonable request.
